# The temporal structure of multiple visuomotor processes in baseball batting: insights from a virtual reality system

**DOI:** 10.3389/fpsyg.2025.1514301

**Published:** 2025-01-30

**Authors:** Naoki Saijo, Takehiro Fukuda, Makio Kashino

**Affiliations:** Human Information Science Laboratory, NTT Communication Science Laboratories, Nippon Telegraph and Telephone Corporation, Atsugi, Kanagawa, Japan

**Keywords:** virtual reality, baseball batting, visuomotor processing, decision-making, ecological validity

## Abstract

Baseball batting is an inherently complex visuomotor task that requires the rapid processing of multiple cognitive-motor computations within a split second. The key components of these computations include the sense of timing, swing decision-making, and swing adjustment. Although each of these components has been studied independently, few studies have addressed their temporal integration. As such, we explored the temporal structure of visuomotor processes in baseball batting using a virtual reality (VR) batting training system. The VR system simulated a mixed sequence of fastballs and breaking balls in which participants were instructed to swing at strikes or take at balls, thus mimicking a real pitcher-batter scenario. The sequence also included pitches where the ball became occluded midway, requiring the participants to maintain accurate swings. Twenty-three batters from a professional Japanese baseball team participated in this experiment. They exhibited the ability to adjust swing timing based on pitch speed, make swing decisions based on strike/ball discrimination, and adjust swing trajectories according to the ball plate location. However, we observed performance deterioration with occluded pitches, particularly in the swing trajectory adjustment, which significantly declined. Swing decision-making showed minor decline, whereas swing timing adjustment remained unaffected. These results indicate that an occluded ball trajectory affects swing adjustment and partially impacts swing decision-making. These findings suggest a temporal structure in the brain’s processing: After the start of pitching, the batter’s brain first handles the computations for swing timing adjustment, followed by swing decision-making, and finally swing trajectory adjustment. Furthermore, the results highlight the potential of VR training systems as powerful tools for elucidating the intricate mechanisms underlying athletic skills.

## 1 Introduction

Athletic skills consist of numerous cognitive-motor functions such as anticipation, reaction, and decision-making abilities (Yarrow et al., 2009; Williams, 2019; Gray, 2021). Athletes demonstrate these cognitive skills during real sports scenarios, achieving exceptionally high athletic performance. Previous studies have focused on isolating the components of these skills and experimentally evaluating the superiority of their individual functions. For example, the superior swing decision-making ability of top-level baseball batters compared with amateur batters has often been evaluated using simplified experimental setups that measure decision-making ability (Gray, 2010; Fadde, 2016; Müller et al., 2016). However, superior individual functions do not necessarily equate to superior overall athletic skills (Dicks et al., 2010). Ideally, the superiority of these component functions should be evaluated in the context of their deployment in real sports scenarios. That said, this has been exceedingly difficult to demonstrate in the past.

Baseball batting is an inherently complex visuomotor task that requires rapid processing of multiple visuomotor computations within a split second. The key aspects of these computations include a sense of timing (Katsumata, 2007; Cañal-Bruland et al., 2015; Nasu et al., 2020), swing decision-making (Gray, 2010; Muraskin et al., 2015; Fadde, 2016), and swing adjustment (Gray, 2002; Higuchi et al., 2016). Although prior studies offer valuable insights into the individual elements of batting skills, they often fail to address how these components are integrated during gameplay. Batting is not merely the sum of isolated processes; it involves the simultaneous and sequential coordination of multiple visuomotor processes.

Virtual reality (VR) systems have the potential to be powerful tools for training and evaluating the component functions of athletic motor skills in realistic settings (Michalski et al., 2019). VR has been applied to various ball sports, such as soccer, basketball, rugby, handball, and tennis, to evaluate and improve motor control, anticipation, and decision-making (Miles et al., 2012). In particular, VR enables researchers to control specific conditions intentionally and observe the batters’ reactions (Isogawa et al., 2018; Takahashi et al., 2019; Ara and Nakamura, 2020; Nasu et al., 2022). Previous research has approached batting mechanics using various methods, including real batting scenarios (Cañal-Bruland et al., 2015; Higuchi et al., 2016) and simulated environments (Gray, 2002; Nakamoto and Mori, 2012; Muraskin et al., 2015). However, these methods often have limitations in terms of experimental control. For instance, in real batting scenarios that use shutter goggles for occlusion tasks, there is a safety risk that limits the range of ball trajectories that can be tested (Higuchi et al., 2016). Likewise, tasks that rely on button presses, rather than actual swings, fail to capture the full complexity of the motor processes involved in batting (Muraskin et al., 2015). Additionally, purely virtual systems might not accurately replicate the pitcher’s delivery motion, which is critical for realistic decision-making during batting (Gray, 2002; Wilkins, 2024).

In contrast, the recent VR training system overcomes these limitations by closely replicating the real pitcher-batter matchup while eliminating the risks associated with physical occlusion. For example, the system that integrates radar-measured ball trajectories with realistic pitcher delivery motions (Isogawa et al., 2018; Takahashi et al., 2019) allows batters to respond naturally in a controlled environment. This capability facilitates the investigation of complex tasks without oversimplifying them, making it possible to explore the dynamic interplay of visuomotor processes in a controlled yet realistic environment.

Our primary goal was to examine the temporal structure of multiple visuomotor processes involved in baseball batting. Using a VR system to recreate pitcher-batter matchup scenarios, we investigated several aspects of batter reactions to various pitches such as sense of timing, swing decision-making, and swing adjustment. In addition, inspired by previous research employing visual occlusion tasks (Fadde, 2009; Higuchi et al., 2016; Müller et al., 2016), we introduced conditions in which the pitch trajectory was selectively occluded midway while preserving all other visual cues—conditions that cannot be achieved in real-life settings—to examine the constitution of visuomotor skills in batters. This approach enabled us to observe subsequent behavioral changes in batters, providing insights into the reliance on continuous visual information for different aspects of batting performance. By analyzing the differential impact of occlusion on batters’ reactions, we aimed to elucidate the temporal structure underlying these processes in baseball batting.

## 2 Materials and methods

### 2.1 Participants

Twenty-three male baseball players from a professional Japanese baseball team participated in this study. Their mean age was 24.61 years (SD = 3.95), ranging from 20 to 35 years. Among the participants, 13 were right-handed batters, and 10 were left-handed. All participants provided written informed consent before taking part in the study in accordance with the guidelines approved by the NTT Communication Science Laboratory Ethics Committee.

### 2.2 Apparatus

**Figure 1:**
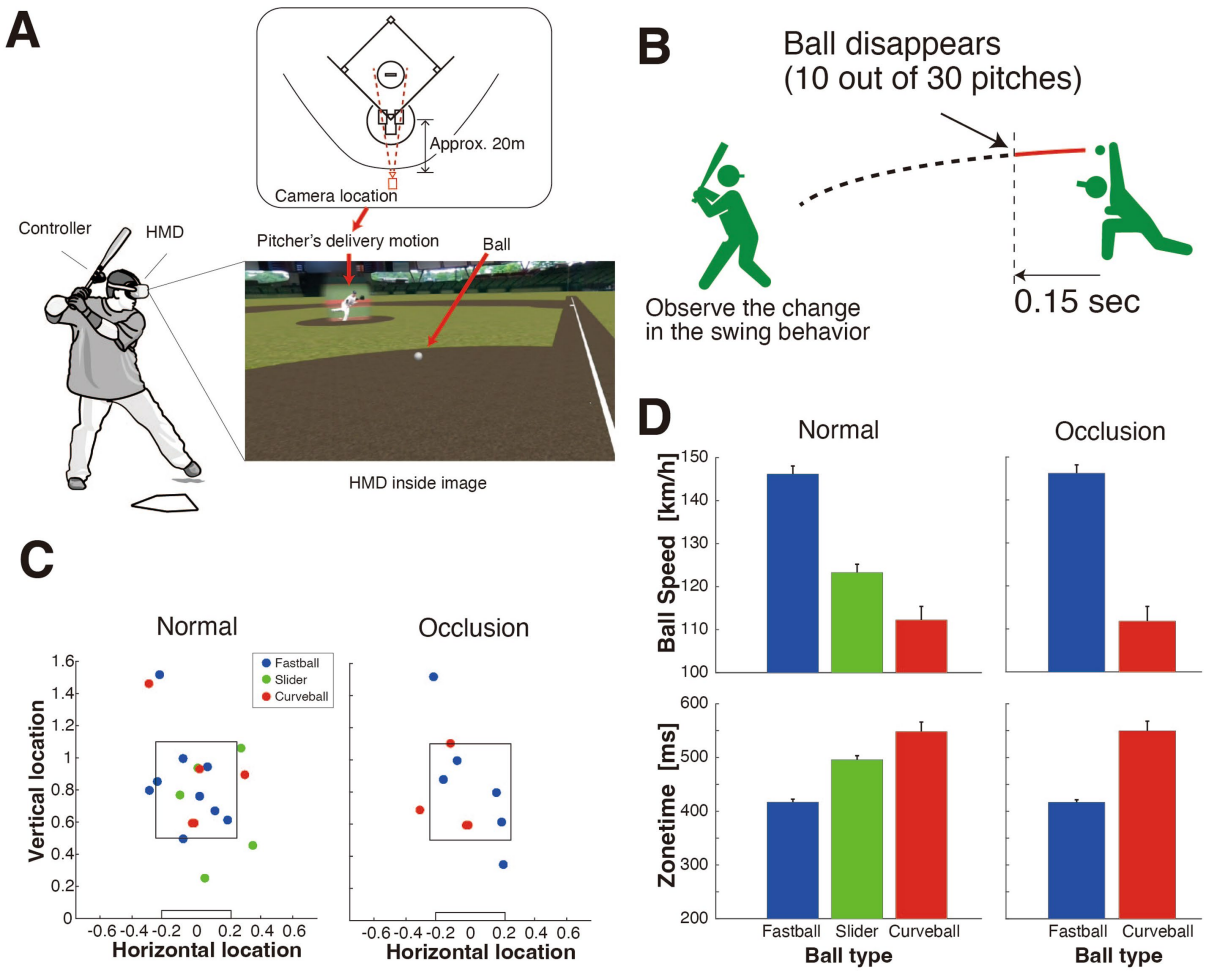
Experimental setup and pitch statistics. **(A)** Experimental setup. The participants wore an HMD and stood on a batter’s plate facing a pitcher in the VR space while holding a bat equipped with a VR controller. The HMD inside image shows the batter’s visual perspective, including the pitcher’s delivery motion and the ball trajectory, as viewed in the VR environment. The pitcher’s delivery motion was captured using a camera positioned approximately 20 meters behind the home plate, slightly offset toward the left batter’s box. **(B)** Schematic illustration of the occlusion condition. In 10 of the 30 pitches, the trajectory was occluded 0.15 s after release. **(C)** Distribution of the ball location (horizontal and vertical) at the point where the ball crossed the front edge of the home plate in the normal (left) and occluded (right) conditions. The rectangular boxes in each plot represent strike zones. The ball type is indicated by green (fastball), blue (slider), and red (curveball). **(D)** Pitching parameters: ball speed (top) and zone time (bottom) for each ball type in the normal (left) and occluded (right) conditions. Error bars represent the standard error.

We utilized “V-Baller” provided by NTT DATA Japan Corporation,^1^ with the hardware consisting of Meta Quest 2. The participants wore a head-mounted display (HMD) and used a controller-equipped bat to perform the batting actions (Figure 1A). The VR content displayed on the HMD was constructed using data from pitching actions and ball trajectories measured during Japanese professional minor league baseball games.

The system records the position of both the controller attached to the bat and the HMD. We derived the primary data used for analyzing batting actions from the position details of the controller in relation to the bat. This setup allowed for precise tracking and analysis of the participants’ batting movements. The HMD displayed the video at a refresh rate of 75 Hz, and the position data of both the controller and HMD were recorded at the same rate of 75 Hz.

The pitching content was developed by shooting the pitcher’s actions from behind the back net during baseball games and measuring the ball’s motion data using TrackMan Baseball, a 3D Doppler radar system (TrackMan, Vedbaek, Denmark). To capture the pitcher’s delivery motion, a camera was placed approximately 20 meters behind the home plate, slightly offset toward the left batter’s box (Figure 1A). The zoom level was adjusted to fit the entire pitching motion within the frame. While this setup provided a practical solution for recording in a stadium environment, the camera’s exact position was approximate due to the constraints of the location. The recorded pitching motion videos were edited and placed on a pitcher’s mound within the VR environment. The ball trajectory was reconstructed in a 3D space based on ball motion data and aligned with the release timing and position of the pitcher in the video. This process created an immersive and realistic pitch simulation.

Thirty different pieces of pitching content were created based on distinct pitching data and presented to batters in random order. Due to experimental time constraints with the professional players, we were limited to 30 pitches per batter. Among these, 10 pitches included an occlusion condition in which the ball disappeared 150 ms after release (Figure 1B), simulating a partial vision condition. The number of occluded pitches was intentionally kept smaller than that of the normal pitches to simulate a scenario in which the batters, who were mentally prepared for normal pitches, were unexpectedly confronted with occluded pitches. This design allowed us to observe their reactions to unexpected occlusion during batting.

The pitch statistics are shown in Figures 1C,D, respectively. We selected the pitching content to ensure that the ball trajectories would be evenly distributed both vertically and horizontally; this selection included some borderline pitches, reflecting the natural variability of real-game scenarios where batters must often make decisions on ambiguous pitches. The ratio of fast to slow pitches was approximately 6:4. We chose fastballs as the fast pitches and sliders and curveballs as the slow pitches. We chose the velocity of the slow pitches to be approximately 15–25% slower than that of the fastballs. The average speeds were 146.18 ± 1.91 km/h for fastballs, 123.28 ± 1.93 km/h for sliders, and 112.22 ± 3.29 km/h for curveballs (Figure 1D). Within the limited number of occluded pitches, we excluded sliders from the occluded pitches to ensure that the latter closely matched the normal pitches in terms of speed and location variability. This decision facilitated meaningful comparisons between the normal and occluded conditions.

### 2.3 Procedure

The participants wore an HMD and stood on a batter plate facing a pitcher in the VR space while holding a bat equipped with a VR controller (Figure 1A). They were instructed to swing at pitches if they judged them to be strikes, just as in a real game, and to refrain from swinging at pitches if they judged them to be balls. It is to be noted that no additional game context, such as pitch counts or situational scenarios, was provided because we focused on the batter’s response to visual information of the pitch, independent of external influences. Additionally, some pitches were programmed to disappear 150 ms after release, and the participants were instructed to respond to these occluded pitches in the same manner as they would to visible pitches, swinging at strikes and not at balls.

To initiate a pitch, the participants had to place a cursor, controlled by the HMD’s movement, on a pitch start button located in front of them for 1 s. Once the pitch sequence began, the participants faced the virtual pitcher and prepared for batting. Simultaneously, at the start of the pitching content, the position data of both the HMD and controller attached to the bat were recorded and continued to be recorded until the end of the content.

After putting on the equipment, the participants received instructions and practiced with 5–10 pitches. Following this practice session, they proceeded to the main experiment, which consisted of 20 normal pitches and 10 occlusion pitches presented in random order. The entire session, from equipment setup to completion, took approximately 15 min.

### 2.4 Data analysis

We processed the position data obtained from the controller attached to the bat using a low-pass filter with a cutoff frequency of 5 Hz to define the bat position. This method was selected based on the system characteristics of our VR setup. This value ensured that the swing trajectory and the associated velocity waveform could be visualized and analyzed consistently with the VR system’s resolution and sampling rate. We determined bat velocity by differentiating the position data over time and defined the magnitude of the velocity vector as the swing speed.

We defined the moment the pitcher released the ball as Time 0 in the pitching content and the swing initiation time as the moment when the swing speed exceeded 4 m/s. This threshold was chosen to reliably capture the initiation phase of the swing while minimizing false detections in our VR setup. We considered a swing to have occurred if swing initiation was detected and the swing speed exceeded 8 m/s within 200 ms after the swing initiation time. We recorded swing occurrences manually to ensure the accuracy of the automated swing detection technique. The results indicate that the automated method is consistent with manual swing records.

We defined the time difference between ball release and swing initiation as the batter’s response time (RT). The zone time was the time taken for the ball to travel from the release point to the front edge of the home plate. The position of the ball was recorded as it passed this edge. We defined the swing position as the bat position relative to the home plate when the bat moved 45 cm in the direction of the pitcher based on the bat position at the start of the swing.

To assess swing adjustments relative to ball location, we obtained unbiased bat positions for each batter under both normal and occlusion conditions. First, we calculated the average horizontal and vertical bat positions for each batter under both conditions. Then, we subtracted these averages from the respective bat positions to obtain the unbiased positions. We compared the resulting unbiased bat positions with the ball locations.

We defined the strike zone as a rectangular area extending from 0.5 m to 1.1 m above the ground and 0.5047 m wide (the width of the home plate plus twice the ball radius of 72 mm), based on the front edge of the home plate. This simplified definition deviates slightly from official rules but we adopted it for ease of analysis. If the ball reached the front edge of the plate in the strike zone, we defined the pitch as a strike.

Statistical analysis involved comparing changes in various parameters under normal and occluded conditions. Due to the limited number of trials per participant, robust within-participant comparisons were challenging. Therefore, to ensure sufficient statistical power, we pooled data across all 23 participants for group-level analyses. We calculated the correlation coefficients between zone time and RT as well as between the ball and bat positions. We compared the slopes obtained from the linear regression analyses in the normal and occlusion conditions using an analysis of covariance (ANCOVA). We assessed the accuracy of strike/ball discrimination using signal detection theory (SDT). Specifically, we calculated the hit rate, false alarm rate, and sensitivity index (d’) for each condition. We then compared these values across the normal and occlusion conditions to evaluate the impact of visual occlusion on decision-making accuracy (DMA).

## 3 Results

### 3.1 Sense of timing unaffected by ball occlusion

**Figure 2:**
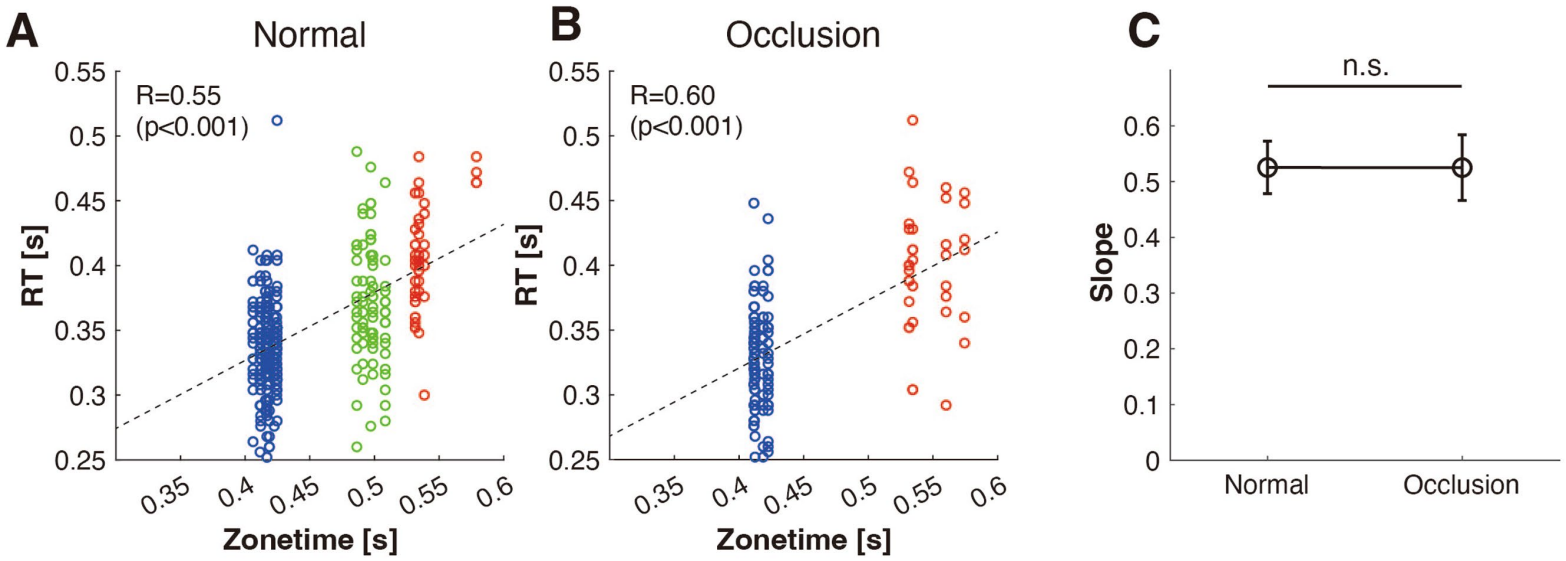
Relationship between response time (RT) and zonetime in the normal **(A)** and occlusion **(B)** conditions, with color indicating pitch type: blue (fastball), green (slider), and red (curveball). **(C)** Comparison of the slopes from the regression analyses in both conditions.

To investigate whether batters could appropriately adjust their swings to different pitch speeds, we examined the relationship between RT and zone time. If the batter correctly identifies the ball speed and adjusts the timing of swing initiation, the RT should change in proportion to the zone time. Figure 2 illustrates the relationship between the RT and zone time for both the normal and occlusion conditions. Figure 2A shows the normal condition, and Figure 2B depicts the occluded condition. In both conditions, RT increased as zone time increased. There were significant correlations between RT and zone time (normal condition: *r* = 0.55, *p* < 0.001; occlusion condition: *r* = 0.60, *p* < 0.001), indicating that the batters adjusted their RT depending on ball speed.

To investigate the effect of occlusion, we compared the slopes of the linear regression in both conditions (the slanted lines in Figures 2A,B). Figure 2C compares the slopes of the regression lines for both conditions. The ANCOVA revealed no significant difference between the slopes (*F*_(1, 437)_ = 1.01 × 10^−5^, *p* = 0.997), implying that the batters’ sense of timing was not affected by ball occlusion.

Notably, in the occlusion condition, the ball could be seen only for the initial 0.15 s from the ball release, but the batters could adjust the swing initiation timing. This denotes that sense of timing is processed in the early phase of the pitch.

### 3.2 Decision to swing at a strike or take a ball partially impaired by the occlusion

To assess whether the batter was making the correct decision to swing at a strike or to take a ball, we evaluated the sensitivity using SDT, which considers whether the pitch was a strike or ball when the batter swung. We calculated the sensitivity index (d’) using SDT, which measures the ability to discriminate between signals (strikes) and noise (balls).

Table 1 shows the results of the strike/ball discrimination accuracy indices for both the normal and occlusion conditions. The numbers in parentheses in Table 1 indicate the total number of strike and ball pitches presented to all 23 participants across the experimental conditions. Compared with the normal condition, discrimination accuracy was degraded in the occlusion condition. Specifically, the hit rate fell from 0.78 to 0.67. Consequently, the sensitivity index (d’) declined from 0.89 in the normal condition to 0.54 in the occlusion condition. This indicates that ball occlusion impacts the correct decision to swing during a strike.

**Table 1:** Strike/ball discrimination accuracy under normal and occlusion conditions.

Condition (strike/ball)	Hit rate	False alarm rate	*d’*
Normal (229/115)	0.78	0.45	0.89
Occlusion (158/70)	0.67	0.46	0.54

Hit rate refers to the proportion of correct swings at strikes, false alarm rate to the share of incorrect swings at balls, and d’ reflects discrimination ability between strikes and balls.

To further investigate the impact of pitch type on discrimination accuracy, we analyzed the data separately for fastballs and curveballs. The results, shown in Table 2, reveal a striking difference between pitch types. For fastballs, the sensitivity index (d’) remained relatively stable, improving slightly from 0.56 in the normal condition to 1.03 in the occlusion condition. In contrast, for curveballs, the sensitivity index substantially declined from 1.62 in the normal condition to 0.21 in the occlusion condition. This decline was accompanied by a reduction in the hit rate from 0.61 to 0.43 and an increase in the false alarm rate from 0.09 to 0.35 under occlusion. This indicates that ball occlusion impacts the correct decision to swing differently depending on the pitch type, with slower pitches, such as curveballs, showing a greater decline in discrimination accuracy compared to fastballs.

**Table 2:** Strike/ball discrimination accuracy under normal and occlusion conditions for fastballs and curveballs.

Pitch type	Condition (strike/ball)	Hit rate	False alarm rate	*d’*
Fastball	Normal (160/70)	0.85	0.68	0.56
	Occlusion (90/47)	0.85	0.51	1.03
Curveball	Normal (69/45)	0.61	0.09	1.62
	Occlusion (68/23)	0.43	0.35	0.21

### 3.3 Swing adjustment affected by the occlusion

**Figure 3:**
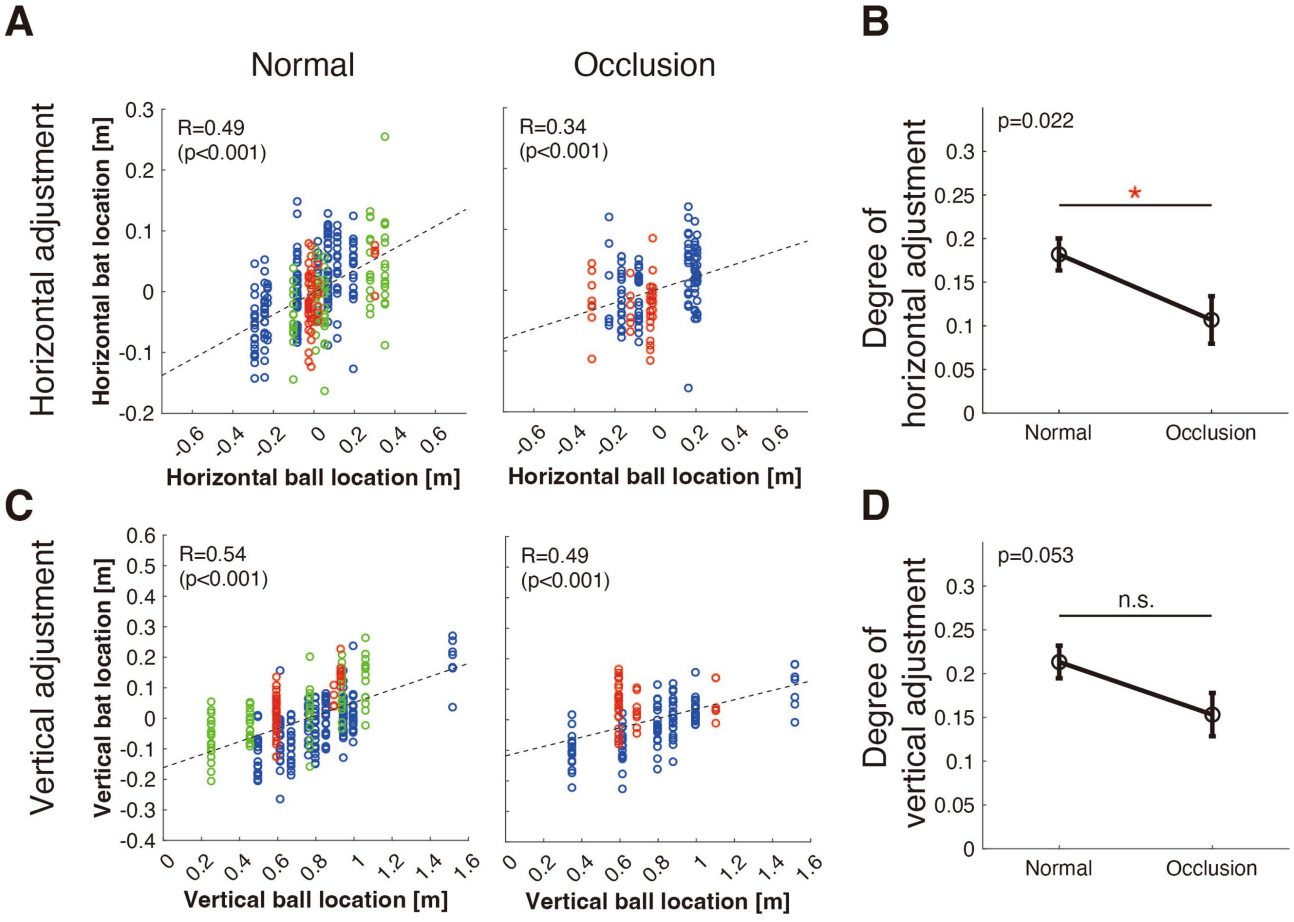
Effect of ball occlusion on swing trajectory adjustments. **(A)** Horizontal adjustment: Relationship between horizontal ball location and horizontal bat location in the normal (left) and occlusion (right) conditions. The color of the dots denotes the pitch type: blue (fastball), green (slider), and red (curveball). Positive correlations indicate that batters adjusted their bat’s horizontal position in response to the ball’s horizontal position. **(B)** Degree of horizontal adjustment: Comparison of the slope of the linear regression lines (degree of horizontal adjustment) in the normal and occlusion conditions. **(C,D)** Vertical adjustment: Shown with the same format as **(A,B)**.

To evaluate the ability to adjust swing trajectory depending on ball location, we analyzed the relationship between ball location on the home plate and the batting position in both the horizontal and vertical directions. The scatter plots in Figure 3 illustrate the relationship between the ball and bat locations for the horizontal and vertical adjustments under both normal and occluded conditions. The slopes of the regression lines indicate the degree of adjustment based on ball position.

For horizontal adjustment (Figure 3A), we noted a significant positive correlation in the normal condition (*R* = 0.49, *p* < 0.001), suggesting that the batters adjusted their swings based on the horizontal position of the ball. In the occlusion condition, the correlation was weaker (*R* = 0.34, *p* < 0.0001), indicating reduced adjustment accuracy. A comparison of the slopes using ANCOVA (Figure 3B) revealed that the slope for the occlusion condition was significantly lower (*p* = 0.022), implying that occlusion adversely affected the horizontal swing adjustments.

For vertical adjustment (Figure 3C), we also observed a significant positive correlation in the normal condition (*R* = 0.54, *p* < 0.001), denoting that the batters adjusted their swings based on the vertical position of the ball. In the occluded condition, the correlation was slightly weaker (*R* = 0.49, *p* < 0.001). The comparison of slopes using ANCOVA (Figure 3D) showed a trend toward reduced vertical adjustment accuracy under occlusion; however, the difference was not statistically significant (*p* = 0.053).

These results suggest that visual occlusion significantly impairs the ability to adjust swing trajectory, particularly in the horizontal direction. This implies that continuous visual information is crucial for accurately tracking a ball and making precise swing adjustments. The reduced accuracy under occlusion highlights the importance of maintaining visual contact with the ball to achieve optimal performance.

## 4 Discussion

### 4.1 Temporal dynamics of visuomotor processes in baseball batting

We aimed to investigate the temporal structure of the visuomotor processes involved in baseball batting using a VR training system. Although the batters’ sense of timing remained unaffected by the occlusion of the ball’s trajectory, both swing decision-making and swing trajectory adjustments were impacted. Specifically, swing decision-making showed partial decline under occlusion, with a decrease in the sensitivity index (d’). Furthermore, swing trajectory adjustments, particularly in the horizontal direction, were significantly impaired by occlusion, as evidenced by the weaker correlation and reduced slope in the regression analysis. These results suggest that different visuomotor processes rely on visual information from distinct phases of the pitch, underscoring the importance of understanding the temporal structure underlying these processes to maintain accurate swing adjustments and effective decision-making in baseball batting.

The differential impact of occlusion on these processes allows us to infer the reliance on visual information after the initial 0.15 s post-pitch release. The sense of timing appeared to be completed using the visual information available before the occlusion, as the relationship between RT and zonetime (slope) was unaffected (Figure 2C). The swing trajectory adjustment relies heavily on visual information beyond the occlusion point, indicating its importance in fine-tuning the swing. Swing decision-making falls between these processes by utilizing visual information both before and after occlusion. This implies a temporal structure in visuomotor processing in which the estimation of timing, swing decision-making, and swing trajectory adjustment are interconnected. Although they may be initiated at different points in time, these processes likely overlap and interact dynamically rather than occurring in a strict sequential order.

For the anticipation of timing, batters can use not only the ball’s trajectory but also the pitcher’s delivery motion. Specific variations in pitcher delivery are linked to different pitch types (Escamilla et al., 1998). Furthermore, when the combination of the pitching motion and the ball’s trajectory is altered in a VR setting, batters struggle to adjust their swing timing accurately (Kimura et al., 2018). These findings denote that batters use the pitcher’s delivery motion to anticipate and fine-tune their swing timing. Thus, it is possible that the anticipation of timing was completed before the ball occlusion took place, as indicated by our results (Figure 2). Batters appear to rely on both the pitcher’s delivery motion (pre-release cues) and early-phase ball flight to fine-tune their swing initiation. Future research could investigate the relative contributions of these visual cues in more detail, which would provide a deeper understanding of how timing anticipation develops at different temporal stages of the pitch.

Systematic changes in the RTs depending on the pitch speed (Fastball vs. Slowball), even in the occlusion condition, suggest that the RT can serve as a valid proxy for evaluating pitch recognition. Previous occlusion-based studies often required participants to verbally identify the pitch type (e.g., Fastball vs. Curveball) after removing visual information (Paull and Glencross, 1997; Fadde, 2006). As Müller and Abernethy (2012) suggested, such tasks primarily engage the ventral vision-for-perception pathway, identified by Milner and Goodale (2006). However, recognizing pitch type during natural batting likely also involves the dorsal vision-for-action pathway. In this context, the observed changes in RT reflect integrated ventral and dorsal pathways because RT is a direct behavioral response to pitch speed that does not rely on explicit verbal identification, allowing the dorsal pathway to contribute to motor preparation and execution in parallel with perceptual recognition through the ventral pathway.

For swing decision-making, batters must determine whether the pitches are within the strike zone while making a swing movement. Expert batters have a heightened ability to discriminate between pitches that should and should not be swung at, particularly when the pitches are within the strike zone (Gray, 2010); they can also anticipate and adjust to fast-moving pitches through enhanced inhibitory control and perception-action coupling (Nakamoto and Mori, 2012; Cañal-Bruland et al., 2015; Muraskin et al., 2015). Based on these findings, the partial decline of the discrimination of a strike or a ball under the occlusion condition in our experiment (Table 1) indicates that the late phase of the ball’s trajectory can be used to establish whether the pitches are within the strike zone and for the inhibitory control of the ongoing swinging motion.

In particular, our analysis revealed that the occlusion condition had a greater impact on the discrimination accuracy for slow pitches, such as curveballs, compared to fastballs (Table 2). While the sensitivity index (d’) for fastballs remained relatively stable under occlusion, it showed a marked decline for curveballs. This suggests that slow pitches, which require batters to rely more heavily on continuous visual input for trajectory discrimination due to their extended flight time, are more susceptible to disruptions caused by occlusion. These findings highlight the differential reliance on visual information based on pitch type, further emphasizing the importance of late-phase visual input in swing decision-making for slower pitches.

In contrast, the fact that the sensitivity index (d’) did not decrease completely under the occlusion condition implies that batters may also use information beyond the ball’s trajectory (e.g., cues from the pitcher’s delivery motion) to aid strike/ball discrimination and inhibitory control regarding swings. While some correct responses could occur purely by chance in a binary task, the d’ values observed here were significantly above zero, indicating the use of systematic information rather than random guessing alone. This denotes that expert batters rely on a combination of visual information from the flight of the ball and predictive cues from the pitcher to maintain DMA, even when critical visual information is partially obscured. Hence, the information on the occluded trajectory of the ball was used for strike/ball discrimination and inhibitory control, resulting in a reduction in swing DMA under occlusion.

The significant degradation of swing adjustment in the occlusion condition points to the role of continuous visual information in fine-tuning swinging actions for batting (Figure 3). This finding aligns with previous research by Higuchi et al. (2016), who demonstrated that occluding the ball’s trajectory 0.15 s after release during a real batting scenario increased variability in the point of contact, particularly in the bat’s longitudinal direction. This indicates that visual information from the latter phase of the ball’s trajectory is critical for fine-tuning the swing. In our results, we observed the differential impact of occlusion on horizontal and vertical swing adjustments, and swing height adjustment was marginally degraded in the occlusion condition, implying distinct processing timelines for horizontal and vertical adjustments of the swing. Gray (2021) highlighted the dynamic nature of visual-motor control in baseball batting, emphasizing the importance of integrating predictive cues (e.g., pitcher’s delivery motion) and real-time visual information from the ball’s trajectory to adjust swing timing and movement. While vertical adjustments, such as those required for estimating pitch drop, may depend more heavily on early-phase visual processing due to the complexity of angular drop speed estimation, horizontal adjustments appear more susceptible to later visual disruptions, such as those induced by our occlusion condition. The significant degradation of horizontal adjustments under occlusion underscores the need for uninterrupted visual tracking to maintain the accuracy of fine motor actions during batting.

Several hypotheses can be proposed to explain why vertical adjustment may occur earlier than horizontal adjustment. First, the biomechanics of swinging may impose constraints that require early vertical adjustments. Second, horizontal adjustments may be delayed because batters can use the bat’s length to compensate for lateral deviations, providing more time to refine these adjustments. Third, from the batter’s perspective, judging the vertical pitch location may be more challenging than judging the horizontal location, necessitating earlier processing to ensure an accurate swing height. Further research is required to confirm these hypotheses.

Our findings regarding differential adjustments and their temporal dependencies provide an opportunity to expand existing models, such as the two-stage anticipation model proposed by Müller and Abernethy (2012), to integrate visuomotor processes more comprehensively. This model emphasizes perceptual anticipation, focusing on the early predictive use of visual cues, such as pitcher delivery motions, to guide decision-making and action preparation. While our findings align with the temporal structure outlined in their model, we extend this framework by emphasizing the role of visuomotor processes in batting. Specifically, our results demonstrate how continuous visual feedback from the ball’s trajectory contributes to late-stage motor adjustments, bridging perceptual predictions with precise motor execution. Swing decision-making likely functions as an intermediate process, where pitch characteristics are evaluated to determine whether to execute or inhibit the swing. This distinction highlights the complementary roles of perceptual anticipation, decision-making, and visuomotor integration in achieving skilled athletic performance.

While this study provides valuable insights into the temporal structure of visuomotor processes in baseball batting, certain limitations should be acknowledged. First, we obtained these findings from professional baseball players, which may have influenced the observed structure of visuomotor processes. At the professional level, players may have developed a refined sequence of information processing because of their extensive training and experience. It remains unclear whether the same temporal structure would be observed in amateur players, who may process visual information differently. This indicates that the observed processing order could be due to advanced training, meaning that specific training protocols may be necessary to develop such a sequence in less-experienced players.

Second, the limited number of trials per participant poses a challenge for examining individual differences in the temporal structure of visuomotor processes during batting. Due to the experimental time constraints with professional baseball players, the number of pitches per session was restricted to 30. This limitation prevented robust within-participant analyses and detailed evaluations of individual temporal structures of visuomotor processes in batting. Future studies should aim to collect more extensive datasets for each participant, enabling the identification and comparison of individual differences in these temporal structures. Such analyses could provide deeper insights into how specific training regimens or natural variability influence individual visuomotor processes, ultimately enhancing our understanding of batting performance at both the group and individual levels.

Third, while the inclusion of borderline pitches in our experimental design is intentional to replicate real-game scenarios, it introduces additional complexity in interpreting the sensitivity index (d’). Decision-making accuracy for borderline pitches is likely to vary across individuals. In addition, umpire judgments of borderline pitches may also exhibit variability in the actual game. These factors can influence d’. If d’ is to be used as a metric for evaluating decision-making accuracy within a specific experimental condition, careful consideration of the design, such as excluding borderline pitches or categorizing them based on consistent criteria, is necessary. This approach could reduce variability introduced by ambiguous pitch judgments, leading to a more precise and reliable assessment of a batter’s strike/ball discrimination accuracy. Despite these complexities, our experimental design successfully captured the impact of occlusion on realistic strike/ball discrimination and provided valuable insights into swing decision-making under game-like conditions. Future studies could explore how to balance ecological validity and experimental control to refine the use of d’ in both experimental and real-world contexts.

### 4.2 Advantages of a VR training system for evaluating athletic motor skills

Our findings stress the importance of continuous visual information throughout the pitch trajectory for accurate swing adjustment and effective decision-making. The VR training system used in this study offers a unique advantage by closely replicating real pitcher-batter matchups while allowing for experimental manipulations such as occlusion of the ball’s trajectory, which would be impossible in a real batting scenario. This capability enabled us to observe batters’ reactions in an environment that mirrored actual game conditions, thereby providing valuable insights into the temporal dynamics of visuomotor processes in baseball batting.

Our VR system, which is similar to that developed by Isogawa et al. (2018), integrates a pitcher’s real game motion with radar-measured ball trajectories into the VR environment. Isogawa et al. showed that this approach yields swing responses in a VR setting that closely resembles those observed in real games, offering a more accurate representation than 2D displays (Isogawa et al., 2018). In our experiment, batters exhibited responses such as adjusting the timing of their swing initiation according to pitch speed (Figure 2), which parallels the findings from studies that recreated real pitcher-batter interactions (Nasu et al., 2020). These results suggest that the VR system is sufficiently capable of replicating responses observed in real batting scenarios, thereby validating its use in testing batters’ information processing characteristics while preserving perception-action coupling (Wilkins, 2024).

HMDs often lead to the perception of objects being closer than they actually are, particularly with respect to depth perception (Renner et al., 2013). In baseball batting, the accurate perception of ball location is crucial. However, in this experiment, we focused on whether the bat position changed in response to variations in ball location across multiple pitches, rather than on the absolute accuracy of ball location perception for each pitch. In other words, the precision of the perception of ball position for individual pitches is less critical, whereas the relative change in ball location over multiple pitches is more important. In the present study, batters adjusted their batting position in response to changes in ball position (Figure 3), suggesting that the discrepancies in depth perception commonly associated with HMDs are not significant.

Despite the limitations of HMDs and VR systems, the assessment methods of swing timing and trajectory adjustments offer valuable practical applications. For the sense of timing, using RT as a measure provides an intuitive and quantifiable way to evaluate how batters anticipate and respond to varying pitch speeds. Additionally, for swing trajectory adjustments, the slope of the relationship between changes in ball location and bat position serves as a robust metric for assessing a batter’s ability to adjust their swing to the ball’s trajectory. These metrics not only provide clear and action-oriented insights into a batter’s performance but also provide useful tools for coaches and sports scientists. By leveraging these quantitative measures, practitioners can design training regimens tailored to specific weaknesses or areas for improvement, potentially enhancing overall athletic performance.

Finally, VR training systems offer the unique advantage of evaluating the superiority of component functions within a context that closely mirrors real sports scenarios without the need to excessively deconstruct the skills being studied. As mentioned in the Introduction, superior individual functions do not necessarily equate to superior overall athletic skills (Dicks et al., 2010). The challenge is to evaluate these functions in a manner that retains their ecological validity (Holleman et al., 2020), which has been exceedingly difficult to achieve using traditional methods. We demonstrated that VR systems provide an effective solution to this challenge. By allowing us to observe and analyze the interplay between multiple visuomotor processes in a highly controlled yet ecologically valid environment, VR technology enables a more holistic understanding of athletic skills. The results of our experiment support this approach, showing that the VR system can capture the complexities of real-world sports performance while still providing the experimental control required to dissect the underlying mechanisms. This approach also holds significant potential for other fields such as psychology, cognitive science, and neuroscience, where the integration of ecological validity with experimental rigor has been a persistent obstacle. VR systems offer a powerful tool to address these issues, enabling more realistic and contextually relevant studies that bridge the gap between laboratory research and real-world applications.

## 5 Conclusion

This study revealed the temporal structure of visuomotor processes in baseball batting, emphasizing the distinct reliance on visual information at different stages of a pitch. Using a VR training system, we found that swing timing is determined early in the pitch, whereas swing decision-making and adjustments, particularly in the horizontal direction, depend on continuous visual input throughout the pitch. The observed performance degradation in the occlusion condition underscores the critical role of uninterrupted visual information in precise motor actions and decision-making. The VR system facilitated the simulation of realistic sports scenarios while maintaining experimental control, facilitating a comprehensive understanding of complex athletic skills without excessive simplification.

In conclusion, this study enhances our understanding of the temporal dynamics of baseball batting and demonstrates the versatility of VR technology in studying complex cognitive-motor tasks. Future research should continue to explore broader applications of VR systems in various fields to gain deeper insights into human performance.

## Data Availability

The raw data supporting the conclusions of this article will be made available by the authors without undue reservation.

## Footnotes

^1^ https://v-baller.com/
